# Supplementary figures and images for: A real-time feedback system stabilises the regulation of worker reproduction under various colony sizes
Source: PLoS Comput Biol. 2023 Mar 24;19(3):e1010840. doi: 10.1371/journal.pcbi.1010840 (PMC10075462; doi:10.1371/journal.pcbi.1010840)

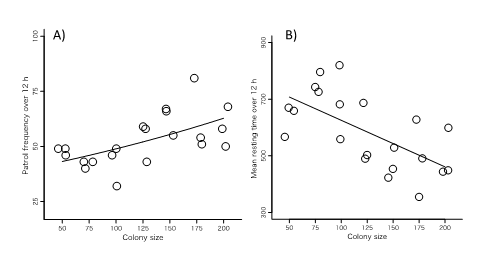

Supplement: S1 Fig — (TIF) [file pcbi.1010840.s001.tif]

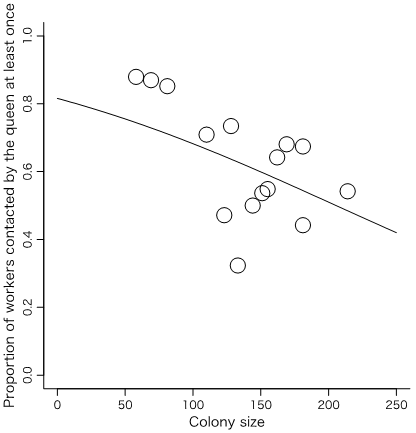

Supplement: S2 Fig — (TIF) [file pcbi.1010840.s002.tif]

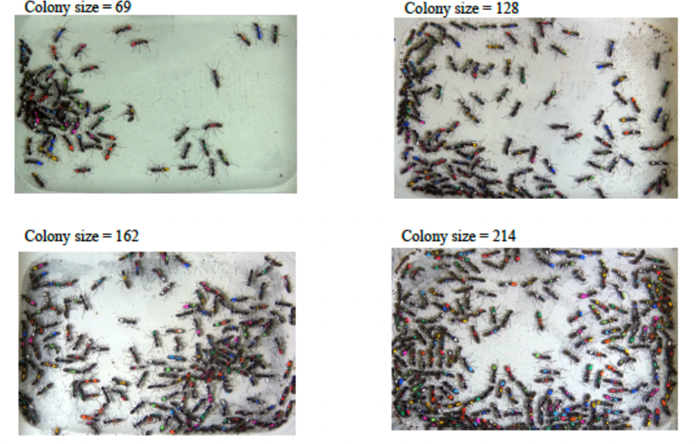

Supplement: S3 Fig — (TIF) [file pcbi.1010840.s003.tif]

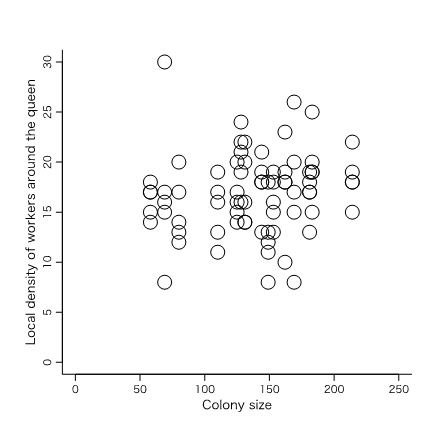

Supplement: S4 Fig — (TIF) [file pcbi.1010840.s004.tif]

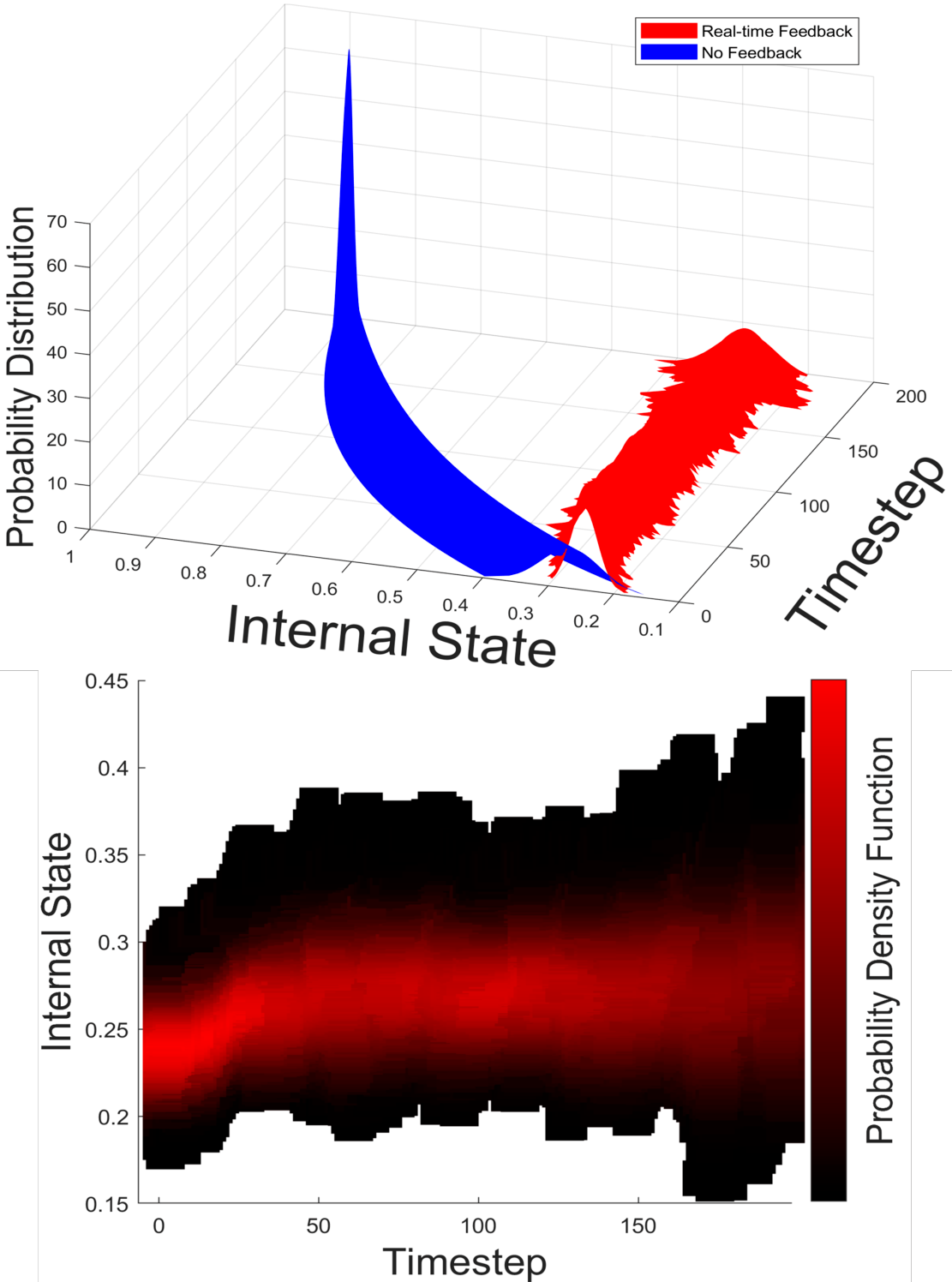

Supplement: S5 Fig — (Top) The probability distribution of workers’ internal states over time in a colony with 120 workers with (red) and without (blue) real-time feedback. The average value increases from 0.2473 to 0.2888. (Bottom) The variance is greater at this colony size. This shows a decrease in the effectiveness of the queen’s patrol behaviour at larger colony sizes. (TIFF) [file pcbi.1010840.s005.tiff]

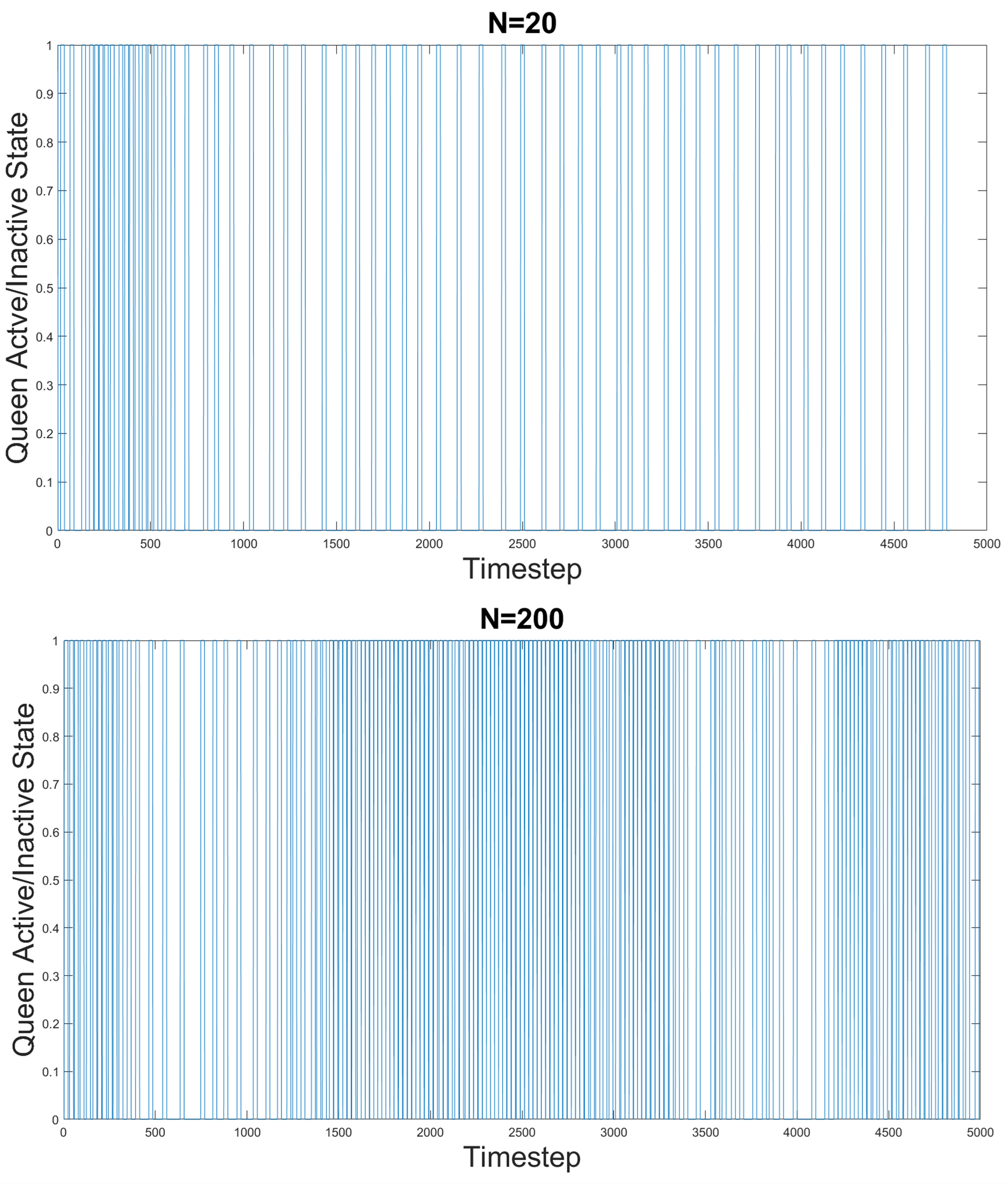

Supplement: S6 Fig — The activity cycle of the queen changes with colony size. As the colony size increases, the rest time of the queen decreases. This increases the frequency of patrol for the queen at larger colony sizes. Here this is seen as clusters of blue lines, with more clusters when N = 200. (TIF) [file pcbi.1010840.s006.tif]

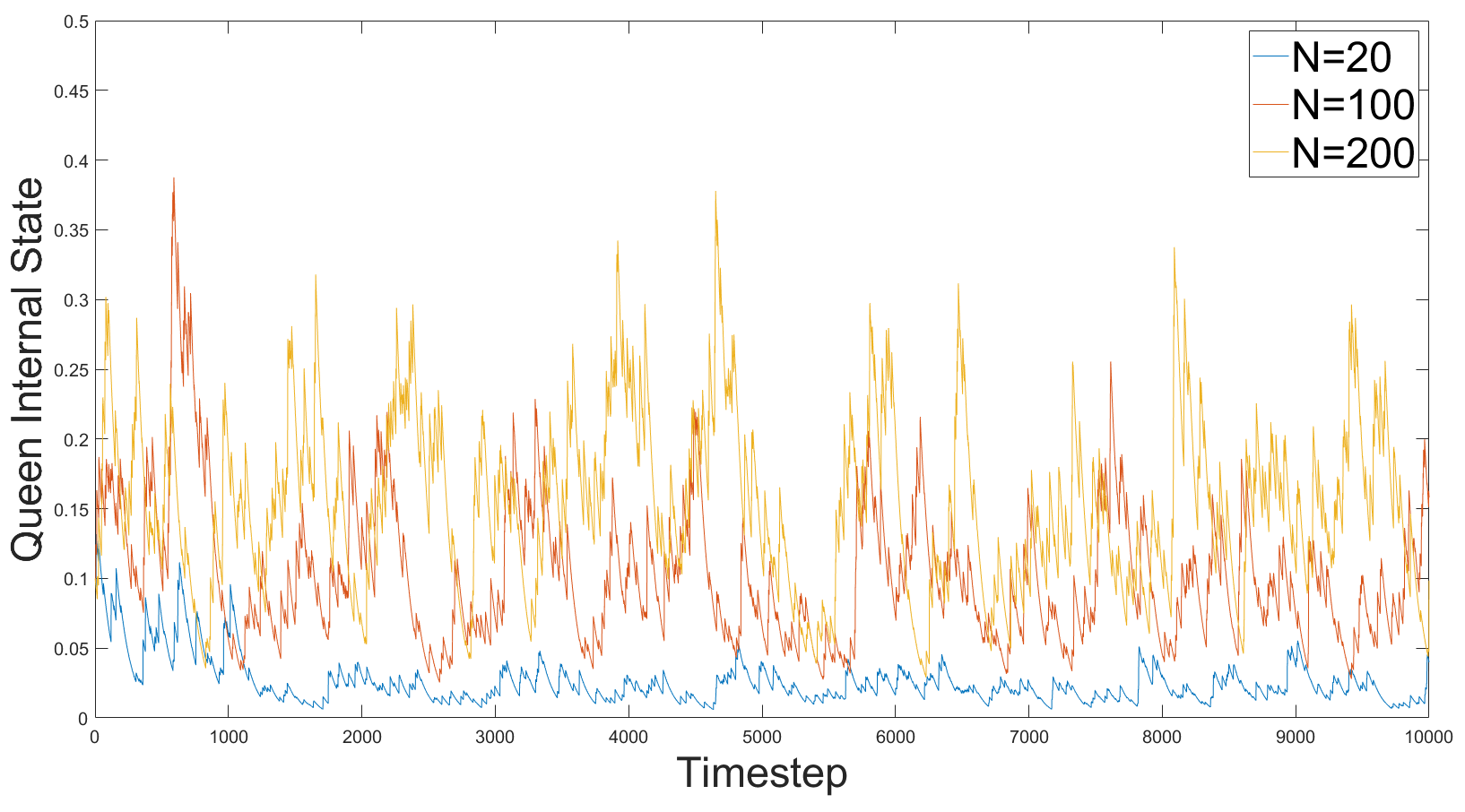

Supplement: S7 Fig — The queen’s internal state changes over time and is coupled with the internal state of workers. Workers in a larger colony have a higher average internal state, causing the internal state of the queen to increase with colony size. (TIF) [file pcbi.1010840.s007.tif]

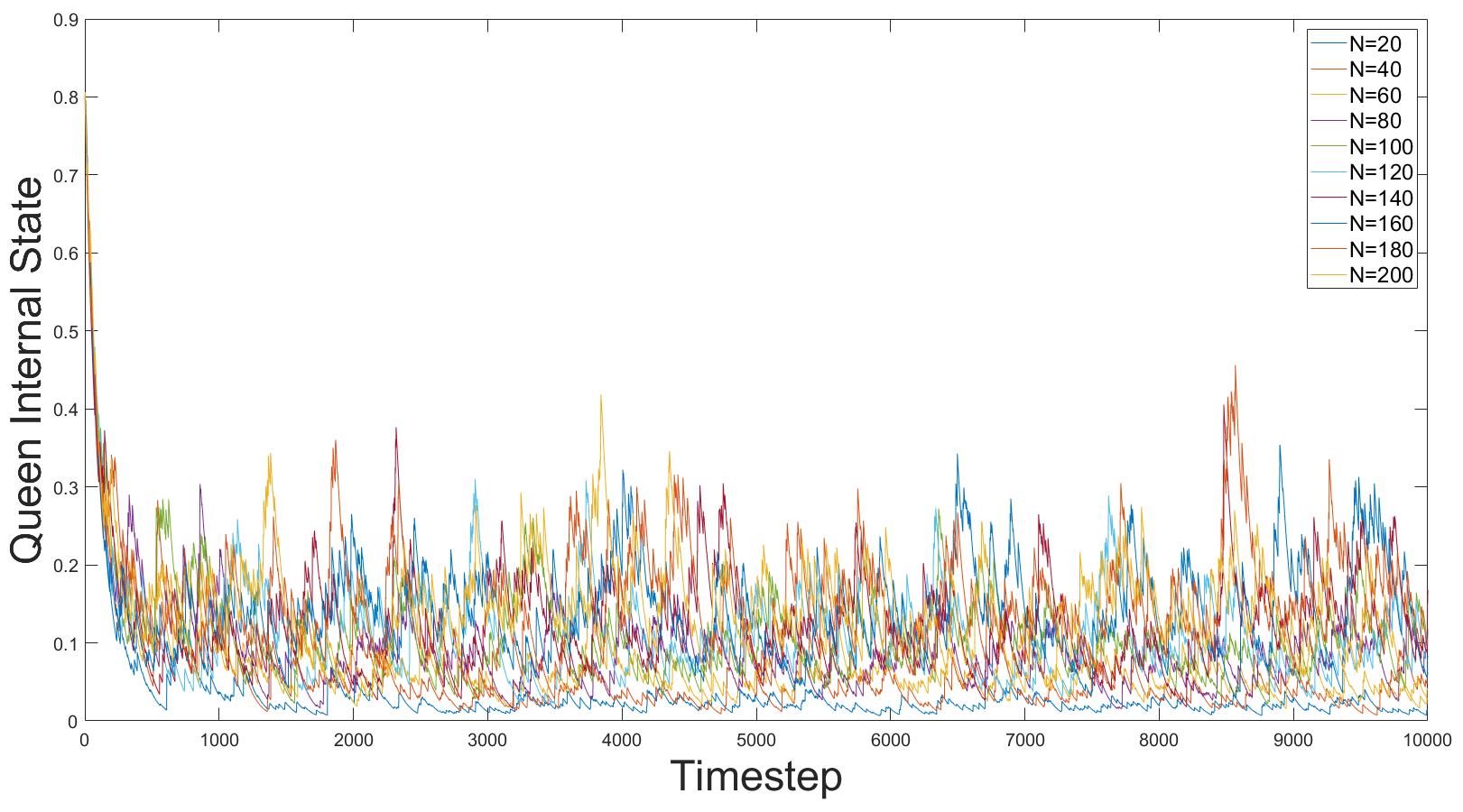

Supplement: S8 Fig — The initial value of the queen’s internal state does not affect the dynamics of the system. Given a higher initial value, the queen’s internal state returns to the normal range observed in the previous figure, with larger colony sizes causing an increased internal state as before. (TIF) [file pcbi.1010840.s008.tif]

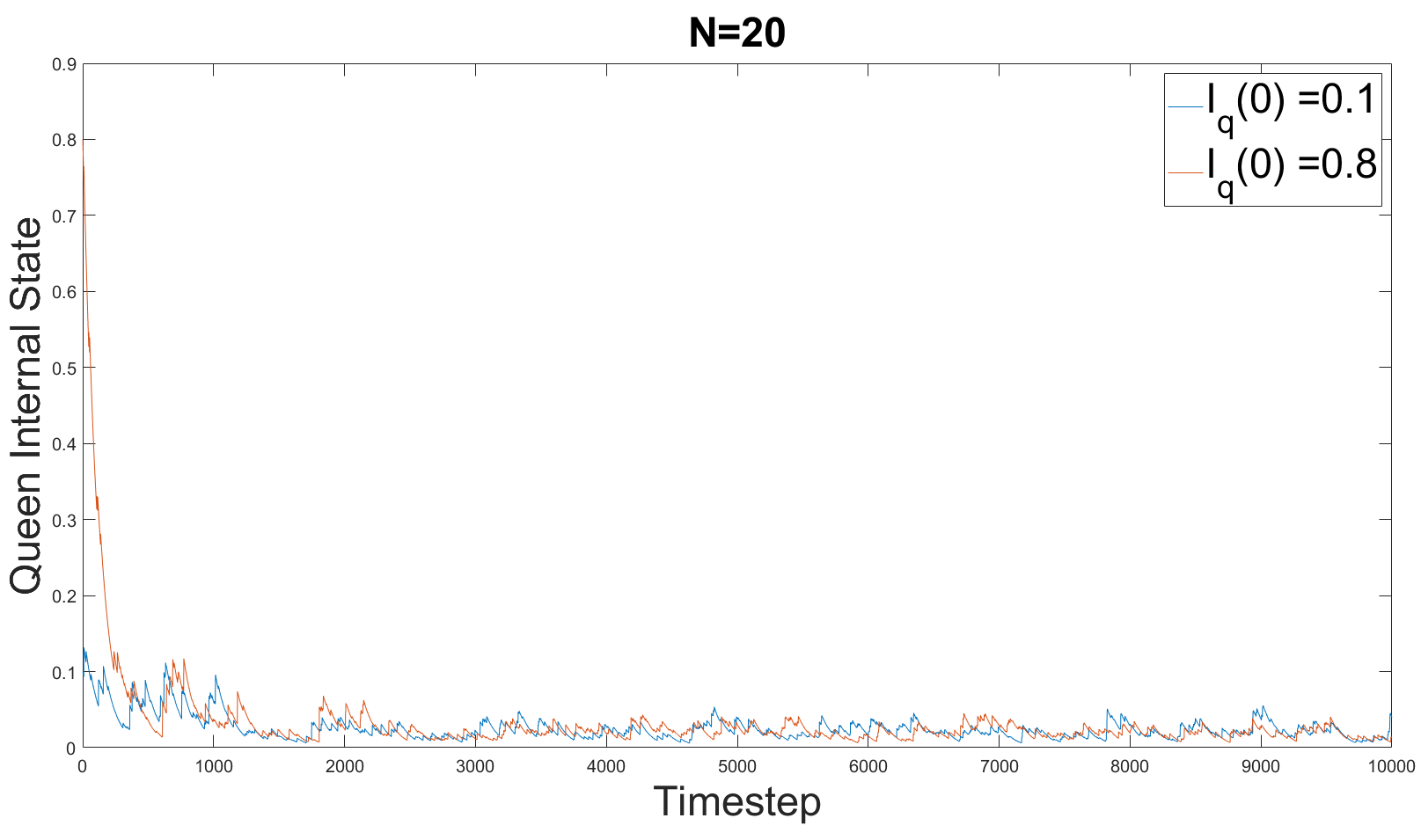

Supplement: S9 Fig — Comparing the internal state of the queen for the same colony size with different initial values, we find that there is a convergence in the queen’s internal state after approximately 400 time steps. This shows that the initialisation of the queen’s internal state does not affect the dynamics. (TIF) [file pcbi.1010840.s009.tif]

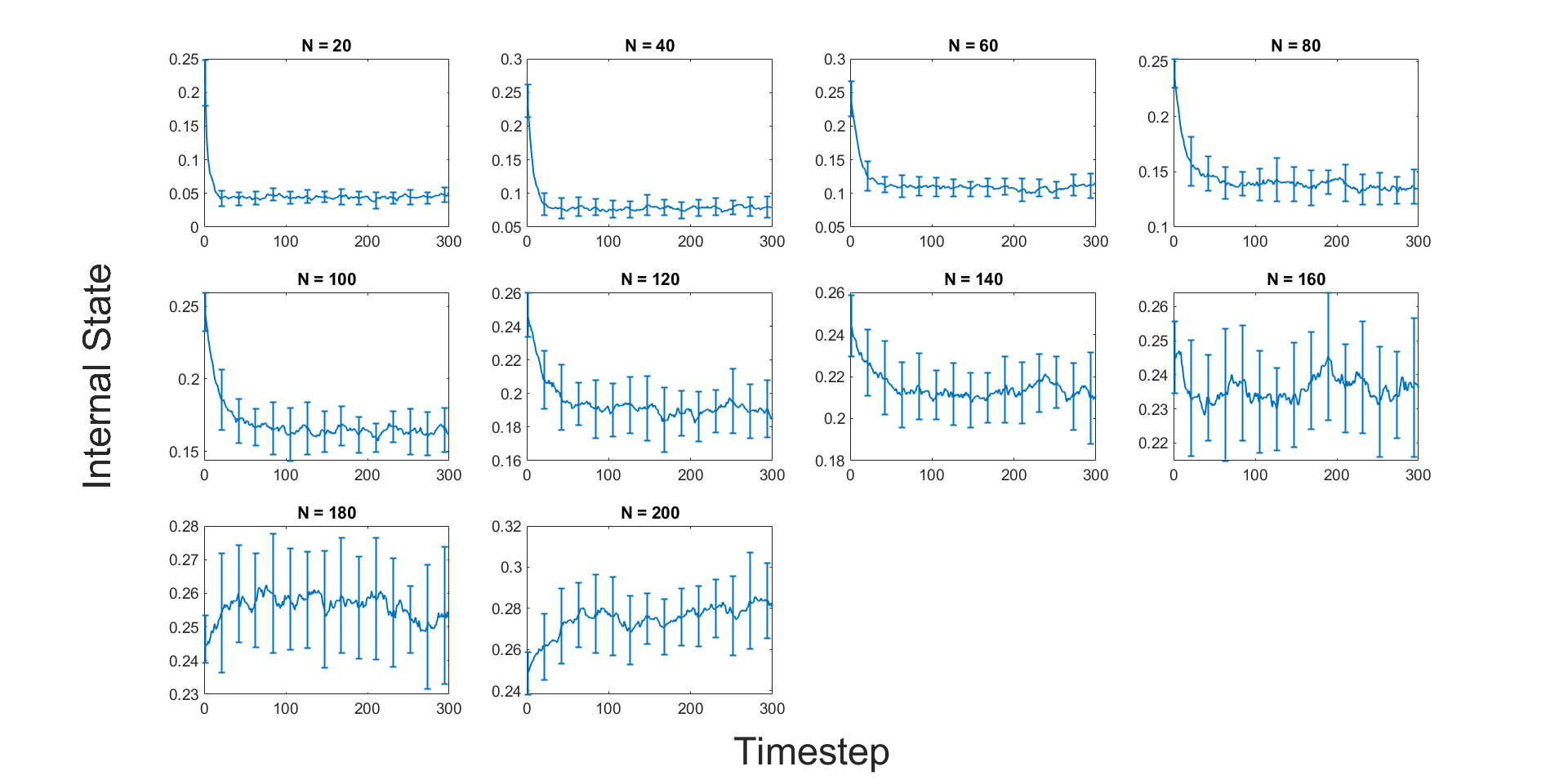

Supplement: S10 Fig — By running the simulation for a consistent period of time for each colony size, we guarantee a steady state for each. With this, we see greater control by the queen over the internal state of workers. There is still weakening in the effectiveness of the queen’s patrol behaviour but the reversal of the suppression occurs much later at the largest colony sizes. (TIF) [file pcbi.1010840.s010.tif]

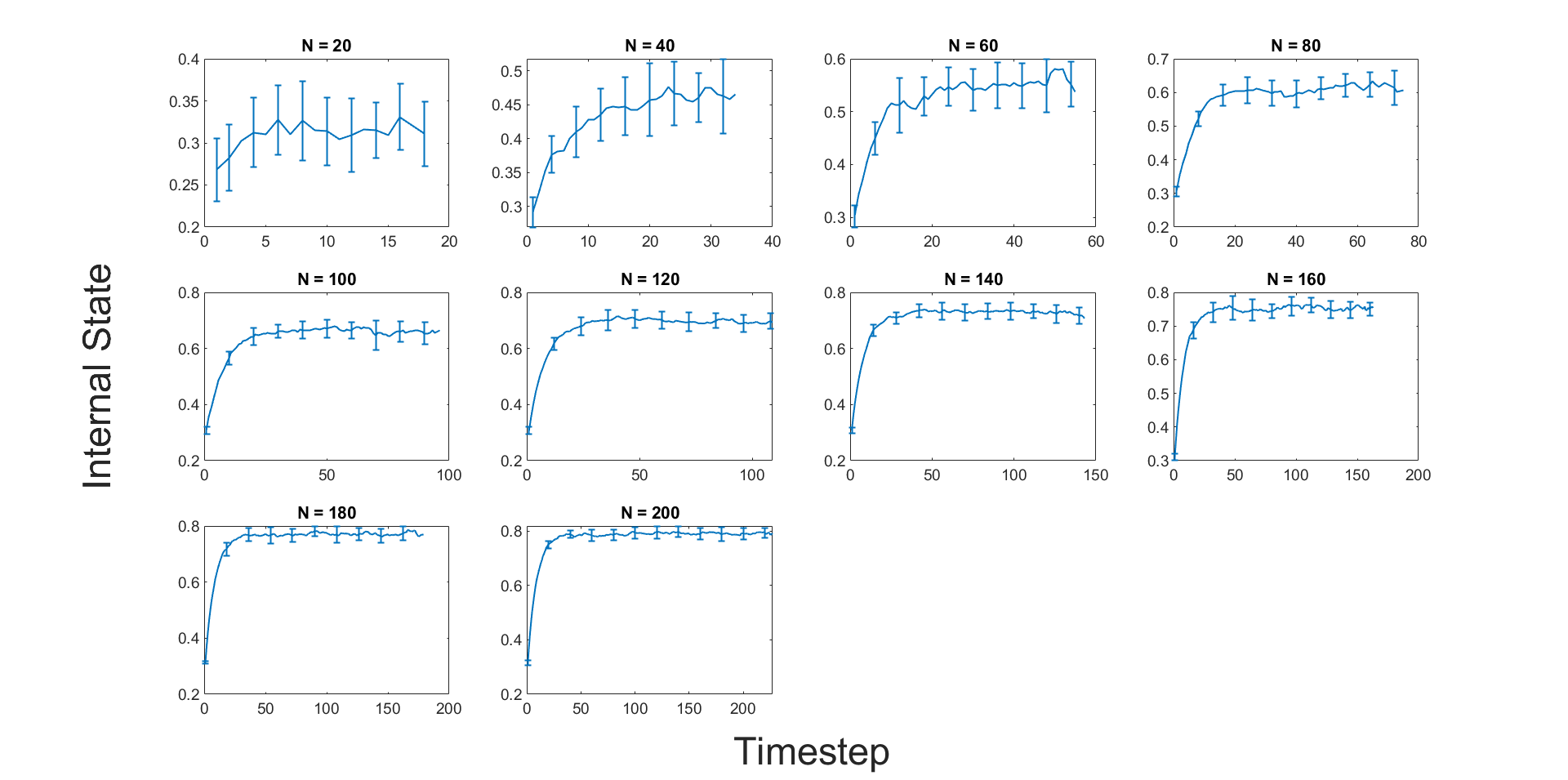

Supplement: S11 Fig — By increasing β and γ, the rate that workers develop their internal state, we showed that the queen has weaker control over the reproduction of workers. Loss of control begins even at smaller colony sizes such as N = 40. (TIF) [file pcbi.1010840.s011.tif]
