# Supplementary material for: A real-time feedback system stabilises the regulation of worker reproduction under various colony sizes
Source: PLoS Comput Biol. 2023 Mar 24;19(3):e1010840. doi: 10.1371/journal.pcbi.1010840 (PMC10075462; doi:10.1371/journal.pcbi.1010840)
Supplement: Attachment — Submitted filename: PLOS_CB_Ver.2.pdf [file pcbi.1010840.s012.pdf]

Title: A real-time feedback system stabilizes the regulation of worker reproduction under various colony size
Author: Simeon Adejumo, Tomonori Kikuchi, Kazuki Tusji, Kana Muruyama-Onda, Ken Sugawara, Yoshikatsu Hayashi

In this manuscript, the authors proposed a mathematical model that simulates the contact dynamics between queen and workers of ant, Diacamma, and numerically explored the regulation mechanism of worker-reproduction rate in a colony, with varying system size (i.e., the number of workers) as a control parameter.

The idea of the size-dependent regulation of worker-reproduction rate through the feedback mechanism has previously been discussed by several researchers including the present authors. However, as mentioned in this manuscript, most of these discussions have focused on the effect of spatial-density increase accompanied by the increase in the number of workers.

The basically new aspect of the present study is that the authors focused not on the number density-increase effect but on the spatially expanding effect by keeping the density of workers in each colony, and confirmed that the pure spatially expanding effect (removing the density increasing effect) causes the negative feedback to regulate the worker-reproduction rate.
Furthermore, they also found the breakdown of the feedback mechanism when the spatial size exceeds a critical one, then, the stage of colony shifts from the “ergonomic stage” to the “reproductive stage”.

In these respects, I consider that the present study succeeded in showing new aspects of the regulating mechanism of the worker-reproduction rate which is one of the basic issues in the research of eusocial insects, and that the findings in this study should be published in PLOS computation biology.

However, in the present form of the manuscript, several problems including the description of the numerical model and the explanation for the obtained results

were found, which may disturb potential readers to reach a clear evaluation of the precious outcomes of the present study.
Therefore, before the final decision on the recommendation of the publication, I would like to require the revision of the manuscript according to the following points.

1. Figure S3 is used to demonstrate that the density of workers is kept almost constant independent of the size of the colony. Seeing this figure, I agree that the density in the regions where workers are staying seems to be kept almost constant.
   On the other hand, low-density regions of workers, also, are observed. Considering that the queen patrols over the whole area in the nest,
   I am afraid if it is appropriate, in defining the "density, to ignore the locally low-density regions which decrease with the decrease of colony size.
   If we include these locally low-density regions in the definition, the spatial average of the worker-density increases linearly with the size, which does not fit the basic assumption of the present study.

2. In P3 eq. (2) and eq. (3), delta functions are used to describe the interaction between the queen and workers. But the present form of using delta functions is mathematically wrong. As authors know, integrating delta function of variable x becomes 1 when integrated by x (if zero-point of x is included in the range of integration).
   However, in the form of eq. (2) and (3), because authors are focusing on the time-development of I_w and I_q, the integrating variable should not be x, but t. Thus, variable transformation term using Jacobian should be included in equations(2) and (3). Anyway, I, most probably, understand what the authors intended with the equations, and, I recommend rewriting these equations, not in the continuous form of equations, but in the discrete form, like, I_q(t+1)=I_q(t)+ ···. Then, the sensitive problems for the usage of the delta function is simply avoided.

3. In the caption of Fig.2 and in L220 P5, there are phrases;
   "Contact is established when workers are within 5 units of the queen ",

"Each agent was set to be 5 units long".
Here, what does it mean the "within 5 units of the queen"? Does it mean "within the 5-cell distance from the queen", or "within 4 nearest cells surrounding the queen or at the queen's cell"
Also a more elaborate explanation for "Each agent was set to be 5 units long" is required.

4. In L224 P6, the phrase;
   "The queen will not make a contact with the same worker twice in a row".
   Could the authors explain a little more clearly the meaning of this phrase?

5. InTable1 P7;
   "Values of N are set as 20,40,60 ,………" , and the value of L is mentioned as "100(when N=20)"
   However, if the density of system is set unchanged with the increase of L, value of L should be increased with the square root of N. That is, because L is set L=100 when N=20, L should be L=141.2 when N=40 and L=173.2 when N=60 etc…. In simulations, what values of L were set when the precise value of L is not an integer?

6. InTable1 P7;
   Are the values of I_q and I_w those of initial condition? If so, they should be described as I_q(t=0) and I_w(t=0).

7. What is the boundary condition of the gird simulation?
   Periodic condition or another?

8. In P4 and Figure 7 and 8, for me, the definition of CRQW, CRWQ and CRWW sounds unclear. If it is not my serious misunderstanding, the total number of encountering events per unit time between the queen and workers seems to be the same both for the queen and workers. So, I, first, guessed that the origin of the difference between CRQW and CRWQ just comes from the difference in the

contact rate per individual considering that the number of the queen is only one whereas the number of workers is larger. However, after that, I realized that this interpretation contradicts with initial stages in Fig.7 where the blue circles are drawn above the black circles. Moreover, in.Fig.8, for the contact rate CRWW per individual, is explicitly described as "per workers".

Because of this situation, with an understanding of the authors' intent included in this study, the model equations, and the outcomes except for fig.7 and 8, I still have not succeeded in completely evaluate the present study.
Therefore, I would like to require the authors a more elaborate explanation for the definitions of CRQW, CRWQ, and CRWW.

9. It is very interesting to see the authors' scenario for the shift from the "ergonomic stage" to the "reproductive stage" according to the present results.
However, I still wonder whether the realization of the shift from the "ergonomic stage" to the "reproductive stage" strongly depends on the parameter in the simulation or not.
For example, if the values of delta, alpha and/or kappa, are set larger than the present ones, the negative feedback mechanism seems to work more remarkably, then, does the shift from the"ergonomic stage" to the"reproductive stage" still occur? Or, are all values set in the simulation supported by reality? .
