# Supplementary material for: A real-time feedback system stabilises the regulation of worker reproduction under various colony sizes
Source: PLoS Comput Biol. 2023 Mar 24;19(3):e1010840. doi: 10.1371/journal.pcbi.1010840 (PMC10075462; doi:10.1371/journal.pcbi.1010840)
Supplement: Attachment — Submitted filename: Reviewer Response.docx [file pcbi.1010840.s013.docx]

# General Comments

We would like to thank the reviewers for their comments on the manuscript and appreciate the feedback given. The fact that the reviewers acknowledge that our model yields interesting results are appreciated. Overall, we think that based on the reviewer’s comments, we could clarify the novelty of our study in the field of computational biology. By applying the engineering concept of feedback control, we showed the effective mechanism of ‘patrol behaviour’ of the queen suppressing the internal states of workers under the constant density of workers.

We also recognise that the section of our manuscript on the contact rates was hard to read for all reviewers. We worked to make the exposition as straightforward as possible. To answer questions from Reviewers 1, 2, and 3 about the sampling at the steady states and the transition point of patrol efficiency, we ran a few new agent-based simulations and added a new dataset to the Supporting Information. Comments from Reviewer 2 have been especially useful in giving line-specific feedback on where the writing could be improved, or concepts better explained for a wide range of readers in biology. We thank Reviewer 3 for identifying the novel biological context in our study.

We hope that you will find our responses sufficient for our paper to be published. As noted by reviewer 3, we also hope the strengthened manuscript helps potential readers reach a clear evaluation of the interesting outcomes of our study and is found to be worthy of being published in this esteemed journal, PLOS computational biology.

# Response to Reviewer 1

We thank reviewer 1 for the feedback and would like to address all the concerns regarding the issues discussed for the main contribution, methods, and presentation in this paper.

**Comment 1** - Issues with the main contribution

“The authors present a new mechanism that they indicate to be superior to the mechanism presented in previous literature. The previous method consisted of the queen regulating her patrolling behaviour as a function of the ant density. The authors say that when the colony size increases, the ant density remains constant, therefore it cannot be used by the queen to regulate her behaviour. However, I do not see how the new mechanism will produce results qualitatively different from the old mechanism.”

“The new mechanism shows that when the colony size increases, the queen is less effective in patrolling, thus leading to a phase transition of the colony where some workers become fertile. This is the only result that the authors use to indicate that their method is correct.”

“I would expect the ‘old’ mechanism to lead to the same qualitative results, in fact, when the queen regulates her patrolling behaviour as a function of ant density and the density is constant, then the queen will put constant patrolling. When the colony size increases, the constant patrolling (which was effective in small colonies) will now become less effective leading to the same phase transition.”

“The authors need to provide a clear comparison between the previous mechanisms and the new mechanism and must bring convincing arguments to explain why one is better than the other. This comparison is not present and the arguments pushed forward are not convincing.”

**Comment 1 Response**

The previous method, in this case, would be the numerical simulation performed by Sugawara which did not include agent-based modelling. This means that the ant density (number of ants for a given space) was not defined in the previous analytical model. Experimental work by Gordon 2021[^1^](https://docs.google.com/document/d/1lQ9XFYXs0Uiyx-6kAr6Sie12st-BeXj1J_dVcBcoKgo/edit#bookmark=kix.6y36d45ip0hm) has looked at how ant density regulates worker behaviour. But these do not look at the patrol behaviour of the queen nor investigate the suppression of worker ovary development, especially using an agent-based simulation.

Thus, in this study, we aim to propose a proximate mechanism which can work under constant density. To this end, we used agent-based simulations to include the spatial degrees of freedom to incorporate the effect of feedback loops through internal states under the constant density of workers. The density is kept constant in recognition of its importance in regulating behaviour. We propose an additional pathway for changes in worker behaviour. Through the altering of internal states. Previous work by Modlmeier et al 2019[^2^](https://docs.google.com/document/d/1lQ9XFYXs0Uiyx-6kAr6Sie12st-BeXj1J_dVcBcoKgo/edit#bookmark=id.ci3v0redkdl3) has shown that despite differences in density, workers maintain interaction rates, meaning that density is not the only cue needed for regulating behaviour.

From this, we developed the agent-based simulation to allow us to (1) simulate realistic experimental conditions where local density fluctuation and excluding volume effect can be taken into account and (2) validate the internal states of workers (effectiveness of the patrol behaviour of the queen). Essentially, the effectiveness of the increased patrol behaviour in terms of stability (convergence of the average internal state).

This is a misunderstanding caused by the lack of clarity in our writing, as described by the reviewer. We worked to make sure this comes through clearly.

Reviewer 1 suggests that a mechanism based on the ant density and our current method would have qualitatively similar results. This is true only for the decrease in the effectiveness of the queen’s patrol. Based on the reviewer's description, keeping the density the same would also mean the queen patrols at the same rate. Our results show that there is an increase in patrol behaviour, but despite this, there is a decrease in the effectiveness of the queen’s patrol. Therefore, multiple results were used to indicate our method was correct, from the increase in the patrol behaviour of the queen (patrol frequency) to the decrease in worker reproduction suppression at larger colony sizes.

In general, there is a breakdown in the feedback loops when the target system becomes too large. Such as the temperature control of a given size room. This remains true in this case, with the increase in colony size beyond a certain point causing a breakdown in the control of worker reproduction. However, this study is the first time ant density has been introduced in an agent-based simulation. So, we cannot make a comparison between the previous mechanism and the new mechanism. In the current study, we tested the hypothesis that the feedback mechanism through internal states can be effective in regulating the internal states of the queen and workers as a function of the colony size when density is constant.

Thus, in our current paper, we addressed biologically plausible conditions (ants walking and contacting in space) and validated the results through the controlled objects (internal states of workers) to answer biological questions using engineering tools (feedback control and simulation) rather than exploring a new control scheme in the field of engineering.

For example, our current approach explains the increase in queen patrol behaviour (see Figure below) when colony size increases. This occurs even when the density is kept the same. The distribution of the internal states of the workers was displayed in Fig 5 in our draft. This was to confirm the effectiveness of the queen's patrol behaviour. Please note that this biologically plausible dataset was presented for the first time in our paper.

Please find the revised parts on Page 3, line 83 describing the previous work by Sugawara that we are building on.

‘Previous work by Sugawara et al. ([34]) theoretically suggested that a feedback mechanism could play a role in the colony size dependant patrol of Diacamma queens. Their model had three assumptions: (1) A worker that has lost contact with the queen for a significant period is released from the inhibitory effects of the queen pheromone; (2) such a worker starts ovarian development and also starts herself emission of the queen pheromone (or other chemicals associated with ovarian development); (3) when the queen, on patrol, comes into contact with a worker who emits such chemicals, the queen increases her future patrol effort according to the chemical concentration she perceives.’

Furthermore, If the queen were to keep her patrol behaviour the same due to constant density, the loss in efficiency would likely be greater than that seen in our results. We believe that, overall, this would decrease the accuracy of our model.

^1^Deborah M Gordon, Movement, Encounter Rate, and Collective Behavior in Ant Colonies, Annals of the Entomological Society of America, Volume 114, Issue 5, September 2021, Pages 541–546, <https://doi.org/10.1093/aesa/saaa036>

^2^Modlmeier, A.P., Colman, E., Hanks, E.M., Bringenberg, R., Bansal, S. and Hughes, D.P., 2019. Ant colonies maintain social homeostasis in the face of decreased density. Elife, 8, p.e38473.

**Comment 2** - Issues with the methods

“Each simulation runs for the time necessary for the queen to encounter once all the workers. As the simulation can be relatively short, especially for small-sized colonies, and it is far from equilibrium, the initialisation of the parameters can have a particularly relevant impact on the results. I suggest the authors study the system at equilibrium, i.e. to run the simulations for a longer time and/or make a more principled initialisation of the parameters. In particular, my intuition is that the initialisation value of the parameter I_q can have an important effect on the dynamics.”

“The details of the random walk are not presented, however, the type of selected random walk can have decisive effects on the results. I suggest the authors indicate with clarity what is the tested random walk behaviour and that they also test other random walks”

“It is unclear what the authors mean when they say on Line 224 - “The queen will not make a contact with the same worker twice in a row.” If two workers are in a range of 5 units with the queen for some number of timesteps would it mean that the contact alternates between the two workers? Because the queen does not make a contact two times with the same ant in a row but at each step with a different one alternating between the same two ants. Or, instead, do the authors mean that the queen never has a second contact with the same worker throughout a simulation? In the former case, it is not clear what is the rationale behind such a choice, and the latter case will have a big impact on the internal state of the queen and it does not seem a correct design choice. Please explain better what you implemented and why you made that implementation choice.”

**Comment 2 Response**

We understand the importance of the initialisation of parameters, and sampling at steady state and agree that they can impact the results. As we were focusing on the dynamical systems in which there are interactions with the feedback loops, the equilibrium could not be defined, as this concept is more related to a closed system under thermal equilibrium. In our study, we sampled the dataset at a steady state. Parameters of interest such as internal states could quickly converge to a steady state. We hope that by clarifying the dynamics of the system, we showed that the initialisation of the parameters has little effect on the dynamics. Firstly, the purpose of the study is to investigate how effectively the queen can regulate worker reproduction. To do this, the queen must patrol the colony and contact all workers. We then compare the queen's ability to reach all workers across colony sizes to determine changes in effectiveness. We argue that the initialisation of the parameters is principled and consistent across the 50 iterations run for each colony size. Please note that different initial values were used for 50 iterations using the random value generator. By doing so, we avoided the steady states becoming trapped at the local minimum in a free energy landscape.

The initialisation of the internal state of the queen (I_q_) does not affect the dynamics of the system at a steady state. The first figure below shows the internal state of the queen at various colony sizes. Throughout the simulation, the internal state of the queen is lower at N=20 than at larger colony sizes N=100 and N=200. This is because the increased colony size (and decreased efficiency) causes the mean internal state of the worker to be higher in larger colonies as illustrated in Fig. 6 of our results. In the second figure below, we change the initial value of I_q_ to 0.8 rather than 0.1. For all colony sizes, I_q_ drops to similar levels to what is observed in the first figure. The third figure illustrates this more clearly. Here we show I_q_ for N=20 when the initial value is 0.1 and 0.8. Although I_q_ is larger initially when set to 0.8, it returns to similar levels of the 0.1 initialisation after 400 timesteps. This shows that the initialisation of I_q_ does not affect the dynamics at the steady state.

We also ran the simulations for a longer period as suggested by reviewer 1. Using the largest timestep from our original results, we simulated 300 timesteps for all colony sizes. The result showed an increase in the effectiveness of the queen’s patrol behaviour in controlling worker reproduction (refer to the below plot). This strengthens our position that the queen can regulate workers' internal states, especially at lower colony sizes.

In line with the suggestions of reviewer 1, we clarified the movement of the agents in the manuscript. Specifically, we outline the details of the random walk.

Please find the revised part on Page 6, line 228.

‘The queen's movement ([52] [53]) around the nest was based on her temporal behaviour: when she is in the active state, she moves around the space, whereas in the inactive state, she halts within the grid she had moved in. The contact behaviour of the queen depended on these temporal behavioural patterns of active–inactive cycles. In this case, the workers also have active-inactive cycles which determined their movement around the space and were determined a priori.’

‘The movement of the queen and workers around the colony was a simple random walk around the nest space. The next position of the agents is generated randomly from one of 4 directions, North, South, East and West. The movements of the agents are asynchronous, with agents only moving when they are in an active state. The queen walks around the nest space to contact all workers in the colony to suppress their internal states. The duration of the simulation was determined by how long it took the queen to contact all workers at least once. The simulation was then terminated. Various variables were recorded for analysis, including the active and inactive period of the queen and the contacts between the queen and workers.’

We would like to clarify the contact between the queen and the workers. The queen does not contact the same worker twice in a row. The queen could alternate between two ants at each timestep, but as the workers and queen move around the colony, it is unlikely that this would occur. We wanted to make sure to avoid the so-called double counting of contacts.

**Comment 3** - Issues with the presentation

“In Figure 2, it is not clear what the blue circles are; the colour legend shows that states are represented with colours from green to brown but several blue circles are displayed. It is also not possible to understand how the runs will evolve over time. Is the movement of all agents synchronous or asynchronous? What’s their motion and interaction pattern?”

“Figure 3 is missing. I suggest the authors carefully double-check their submission files before sending them to review”

“Section Results - Lines 258-265 - This part of the text pertains to the Methods and not to the Results (and the information was in part already presented earlier). Section Results - Lines 268-273 - This part of the text is difficult to read and it is not clear what is the difference between CRQW and CRWQ. How is the distinction between the two done? and why is it important? Additionally, the text is not correctly organised, as the concepts of contact rates are introduced here and then ignore for a few pages and then the results about them appear much later in the text. This description should go just before the presentation of the results of Fig. 7.”

“Fig. 4 - Unclear what patrol frequency is. Never defined. Please define all metrics clearly in a way that your work is reproducible. Fig. 5 is hard to read and not well presented. Rather than a 3D plot which is hard to visualise and understand, I suggest the authors use a 2D colourmap (with colour shades indicating the values of the PDF).”

“Fig. 6 - “... before stabilising at a higher value.” - Higher than what? Fig. 7 - “during the rest cycles of the simulation.” – It is unclear why the results are presented in distinct groups in relation to the rest cycles. What does this distinction mean? Resting cycles of whom? the queen or the worker? Please specify clearly how you computed these values. It is not clear how this data is extracted from the simulations, which quantity do you report? How do you compute CRQW and CRWQ, is it the same quantity divided by the colony size? If yes, I would use a better name (e.g. CR total and per worker).”

**Comment 3 Response**

We understand now that Figure 2 was not clearly laid out. It was meant to show how the agents looked in the simulation environment with workers who had interacted with the queen in blue. This gave a visual representation of the progress of contacts by the queen in the colony. We changed the colouring to reflect the internal state of the workers shown by the colour legend. The agents move randomly around the simulation space, with interactions occurring when the agents are within five units of each other.

Please find the revised part on Page 9, line 297.

‘A contact is determined when a worker is close to the queen, within the length of 5 units (see evidence of the ant’s morphology Fig.2C). The queen can only contact one worker in each time step. Therefore, we decided that the queen would not contact the same worker twice in a row. This is to decrease the prospect of a worker who has already been contacted recently monopolising contact with the queen despite other workers being in range in a short period of time. When the queen contacts the worker, the internal state of the queen and the worker increase and decrease respectively. The increase in the queen's internal state is proportional to the internal state of the worker, while the decrease in the worker's internal state is constant (approx. a 90% decrease).’

The reviewer mentions that Figure 3 is missing. We were sure to check our submission before sending it for review and are unsure why Figure 3 would be missing during the submission process. We hope that, during resubmission, this does not happen again and all our figures are visible to the reviewer.

The section about the contact rates was not clearly laid out with a disjointed explanation. We rectified this by clearly placing the description of the concepts before the presentation of the results in Fig. 7. CRQW and CRWQ reflect the contact rate of the queen and the contact rate of the worker(s). The contact rate of the queen is the number of workers the queen contacts, while the contact rate of the worker is how many times the worker is contacted by the queen.

The contact rate of the queen is simply the number of contacts the queen has per unit time during the simulation. While the contact rate of the workers is an average of the individual contacts they have received. This average should be close to the contact rate of the queen. The distinction was made because if the contact rate of the queen were to increase at a sufficient rate, then there would not be a decrease in the contact rate of the workers. For example, if there are 10 workers contacted once each, then the contact rate for the queen would be 10, and for the workers, 1. If there are 20 workers, to maintain the same contact rate of the workers, the queen now needs a contact rate of 20. If the contact rate of the queen is 15, then the contact efficiency has decreased. We differentiate between the rest cycles and patrol cycles of the queen to demonstrate that most contact between the queen and workers occurs when the queen is patrolling.

We also replaced Fig. 5 with a combination of the 2D colour map, as suggested by reviewer 1 (see above) and our original 3D plot. We hope that this new figure is more understandable.

The patrol frequency is how often the queen patrols the colony. As the patrol time stays the same, in this case, the patrol frequency was determined by changes in the rest time of the queen. The patrol frequency increases due to shorter rest times by the queen (demonstrated above in previous figures).

Referring to Fig. 6 caption, the reviewer asks, “Higher than what?”. The average internal state of workers at the start of the simulation is approximately 2.5. At smaller colony sizes, the average internal state decreases from this initial value, while at larger colonies, the average increases above the initial value. We made that clearer in the figure caption.

# Response to Reviewer 2

We thank reviewer 2 for their extensive line-by-line feedback. Making sure our work is as understandable as possible for biologists and engineers, we appreciate the suggestions of restructuring our explanations of the concepts and a clear discussion on what the model parameters represent. We made the recommended changes to the paper as suggested by reviewer 2, including adding some of the methods of the experimental data in the “Materials and Methods” section.

We would now like to address the comments made by reviewer 2. Firstly, reviewer 2 suggests:

“the entire model rests on the assumption queens can detect the internal state of worker, but no attempt to support this by referring to other social insect taxa has been made”

“this limitation be addressed in the introduction and some justification given”

We posit that there is at least circumstantial evidence that the queen can detect the internal state of workers (Shimoji et al 2017^3^). The queen can suppress worker reproduction through dominance interactions with workers. The ability to sense worker ovary development is necessary to determine how and when these interactions take place.

Reviewer 2 also suggests:

“Line 23: I think the argument here is that even with chemical signalling, there can potentially be an issue of control as the colony increases in size. If so, I think this could be made clearer as it’s the pivotal point the paper rests on. You could also highlight how important this is, i.e., in very large colonies where they may be millions of insects – how is worker suppression achieved?”

We believe the point to be two-sided. First, the mechanism proposed can control worker reproduction at generally smaller colony sizes. Second, while there is a decrease in control efficiency as the colony size increases, there is still an equilibrium point reached at larger colony sizes, as illustrated in Fig. 6. We clarified this in the figure caption for Fig. 6, see below:

“As the internal states of workers were initialised randomly between 0 and 0.5, the average initial value for all colony sizes is approximately 0.25. At smaller colony sizes (N = 20, 40, and 60), the average internal state of workers decreases as a function of time and stabilises at a point which is lower than the initial value. As the colony size becomes larger, there appears to be a transition (at N = 80 and 100) where there is greater fluctuation in the average values over time. At the largest colony sizes (N = 140 to 200), there is an increase in the average internal state of workers over time. Here too there is stabilisation but at a point higher than the initial value.”

Reviewer 2 notes that:

“Line 182”, “I see now from the methods that the queen only contacts about 80% of the colony – would it make more sense given you know this constraint to update the model so it stops once she has contacted 80% rather than 100?”

We thank reviewer 2 for raising this point. The purpose of the queen’s patrol behaviour is to suppress worker reproduction in the colony. As this would apply to all workers, the fact that we found the queen was only able to contact at least 80% of the workers means that some were able to escape the patrol behaviour of the queen. This is a failure in the queen’s ability to control worker reproduction. The repercussions of this on the results of the current study may be the slight overestimation of the effectiveness of the queen’s patrol behaviour.

However, reviewer 2 raises another point, stating:

“Line 216: In our experience the distribution of ants is not random but concentrated around the queen or brood piles, particularly in small colonies (Casillas- Pérez et al. 2021) – how might such a distribution affect your results, since it should make it easier for the queen to contact as many workers as possible?”

Further stating:

“Line 404: This assumes that ants distribute themselves evenly across nest space though, which I think is unlikely. If a colony concentrates most of its workers in particular chambers or has a stable proportion of workers dedicated to certain task that interact with one another, then encounter rate might be a predictable estimate of colony size.”

We believe this distribution would increase the effectiveness of the queen’s patrol. Especially if the movement of the queen and workers is not random, as reviewer 2 suggests. This means that, on this account, the model may underestimate the effectiveness of the queen’s ability to suppress worker reproduction. Overall, the previous overestimate and underestimate cancel each other out to some extent, leaving the current results as an accurate (but possibly imprecise) way of measuring the effectiveness of suppressing worker reproduction. Furthermore, as the distribution around the queen is constant, it is simpler to have an even distribution of workers in the simulation space, which replicates the distribution around the queen. And by doing that, we removed the cue for using the encounter rate as a reliable signal for colony size. We added a section in the introduction to address this, drawing on previous work by Franks et al^5^ and Tschinkel^6^.

Please find the revised section here on page 4, line 102.

“However, the average individual density for a given space in the ant’s nest may not

change so much even if the colony size changes. Franks et al. ([36]) found that ants

changed the size of their nest space to fit with the colony size, making changes to the

nest as required. Though others ([37]) found that the increase in nest space is slower

than the increase in colony size, the largest variation in worker density occurred due to

seasonal changes. Inter-individual distance also appears to be regulated ([38]). Indeed

in Diacamma the worker density per space in the nest remains almost constant (see the

experimental results in S4 Appendix, S4 Fig in appendix).”

Overall, for the patrol behaviour of the queen, we believe we demonstrated in our simulations that her patrol behaviour should be directly dependent on the internal states of the workers rather than the encounter rate, which can fluctuate locally. Please note that the internal states are ‘target’ values in terms of the feedback control for the patrol behaviour. The density of workers or the colony size is not the target value in the control loops for behavioural patterns. In a future study, it is reasonable to run agent-based simulations which reflect the realistic structure of ant colonies.

Reviewer 2 kindly enquires about the model, stating:

“Line 211: It would be really nice if this section could be given some biological context, i.e. how much of the model here is assumed and how much is based on approximating the biology of the ants. To ensure that the paper is understandable to social insect researchers that are not familiar with such mathematic notations, placing these into biological context would be really helpful, e.g. what is the damping factor attempting to replicate in in the real biological system? I think this is where you could incorporate more about the real-world data you collected and how it has informed your model.”

These relate to points raised by all the reviewers about improving the manuscript's clarity, with reviewer 2 asking for more accessible language for the parameters used in the simulation model. We made sure to add the relevant text explaining the answers to the questions presented here by reviewer 2.

Please find the revised part on Page 7 and Line 261:

“(1 − e) · Iq (t) is a damping factor, where e= 0.01 and is constant. As the reproductive

division of labour enables the queen to be the main producer of offspring in the colony,

there is a compromise between patrolling the colony and laying eggs. As the internal

state of the queen represents the likelihood she will become active, we use the damping

factor to naturally decrease the queen’s internal state over time. This allows the queen to move to a more restful state, where there is minimal patrol, assuming workers have a low

internal state. The second term, α · δ(x_q − x_w) · Iw(t), is an activation factor. The

activation factor acts to increase the probability of the queen becoming active when the

queen contacts a worker with a high internal state. It is also proportional to the number

of contacts with the workers. α = 0.1 and is constant. x_g and x_w denote the positions

of the queen and the worker respectively. The term δ(r) denotes a delta function, i.e., its

value is zero except when the distance between x_g and x_Wi is zero, then δ(r) = 1.”

The variables of internal state, rest time and active time are based on observations and extrapolations of behaviours seen in Diacamma colonies. The relation between the variables is also based on previous work by Kikuchi et al 2008^4^, specifically the decrease in rest time by the queen as the colony size increases.

Assumptions are made based on the internal state of the workers developing over time (linear vs exponential increase in the internal state). These are difficult to measure in real time in actual colonies. The damping factor described in the methods decreased the likelihood that the queen would patrol the colony. This only occurs if there is no contact from workers. Contact with workers increases the probability that the queen will patrol based on how high the internal state of the worker is (i.e., how developed the ovaries of the worker are).

For figure 5, we attempted to clarify the figure by including a 2D colour map with the 3D plot. We also revised the figure caption to reflect this. We also clarified the section discussing the contact rates between the queen and workers. Please find the revised part on page 16, line 402.

“By logging the number of contacts that occurred during the simulation, various contact rates could be calculated. These are the queen contact rate, the per-worker contact rate and the contact rate between workers. The contact rate is defined as the number of contacts per unit time. Therefore, the queen contact rate is the rate at which the queen contacts workers per unit time. While the per-worker contact rate is the average contact rate of a worker in the colony. These values were calculated separately, with contacts being logged for the queen and individual workers. Theoretically, assuming an even distribution of contacts between workers, the per-worker contact rate is equivalent to the queen contact rate divided by the number of workers. However, the per-worker contact rate conveys the contact efficiency of the queen and therefore, the effectiveness of the queen's patrol behaviour.”

“Figure 7 shows various contact rates between the queen and workers as a function of the colony size. Figure 7A shows the overall contact rate for the queen increasing (black line) while the per-worker contact rate decreases (blue line). Despite the increased patrol effort by the queen (shown in Fig.4), the contact efficiency of the queen decreases with colony size. This is due to the insufficient increase in the queen contact rate. Distinctions were made for the contact rate during the rest cycles (Fig. 7B) and patrol cycles (Fig. 7C) of the queen. This was to demonstrate that the majority of the contacts by the queen were made when the queen was patrolling.”

Finally, reviewer 2 suggests:

“Line 443”, “this assumption is a major one”, “Is there any evidence in this species, other ants, or in other social insects (perhaps in honeybees/ bumblebees) that queens can do this?”

In eusocial insects such as Pachycondyla[^7^](https://docs.google.com/document/d/1lQ9XFYXs0Uiyx-6kAr6Sie12st-BeXj1J_dVcBcoKgo/edit#bookmark=id.jr4fqzvuv1p7) and Dinoponera[^8^](https://docs.google.com/document/d/1lQ9XFYXs0Uiyx-6kAr6Sie12st-BeXj1J_dVcBcoKgo/edit#bookmark=id.kffxmuvczykk), the queen uses dominance interactions via physical contact with workers. These interactions maintain the division of labour in the colony. In a future study, we would expand the agent-based simulation further to incorporate an assessment of fitness to clarify the limiting factor for the time invested in patrolling by the queen. Previous works, such as Ohtsuki and Tsuji[^9^](https://docs.google.com/document/d/1lQ9XFYXs0Uiyx-6kAr6Sie12st-BeXj1J_dVcBcoKgo/edit#bookmark=id.5dhkg67i4wmp), have laid the groundwork developing a dynamic game model. Elements of the model could be incorporated into the agent-based simulation.

^3^Shimoji, H., Aonuma, H., Miura, T. et al. Queen contact and among-worker interactions dually suppress worker brain dopamine as a potential regulator of reproduction in an ant. Behav Ecol Sociobiol 71, 35 (2017). https://doi.org/10.1007/s00265-016-2263-3

^4^Kikuchi, T., Nakagawa, T. and Tsuji, K., 2008. Changes in relative importance of multiple social regulatory forces with colony size in the ant Diacamma sp. from Japan. Animal Behaviour, 76(6), pp.2069-2077.

^5^Franks NR, Wilby A, Silverman BW, Tofts C. Self-organizing nest construction

in ants: sophisticated building by blind bulldozing. Animal behaviour.

1992;44:357–375.

^6^Tschinkel WR. Sociometry and sociogenesis of colony-level attributes of the

Florida harvester ant (Hymenoptera: Formicidae). Annals of the Entomological

Society of America. 1999;92(1):80–89.

^7^Ito, F., Higashi, S. A linear dominance hierarchy regulating reproduction and polyethism of the queenless antPachycondyla sublaevis. Naturwissenschaften 78, 80–82 (1991). https://doi.org/10.1007/BF01206263

^8^Monnin, T. and Peeters, C., 1999. Dominance hierarchy and reproductive conflicts among subordinates in a monogynous queenless ant. Behavioral Ecology, 10(3), pp.323-332.

^9^Ohtsuki, H. and Tsuji, K., 2009. Adaptive reproduction schedule as a cause of worker policing in social Hymenoptera: a dynamic game analysis. The American Naturalist, 173(6), pp.747-758.

# Response to Reviewer 3

We appreciate the kind words and potential that reviewer 3 has seen in our work, especially in the context of applied engineering in the field of biology. As with the other reviewers, we appreciate the need to improve the clarity of the writing. In pursuit of this, we took account of the feedback from reviewer 3 and re-written equations 2 and 3 in the discrete form to avoid future confusion. We also increased the clarity of the writing, taking into account all the points of improvement laid out by all the reviewers.

Reviewer 3 enquired about the simulated nest space. Simulations were conducted in the Processing Integrated development environment (IDE). The value of the simulated space was set to the more precise values calculated by the reviewer. Reviewer 3 also enquires about the boundary condition of the simulation. To clarify, the agents were prevented from going outside of the virtual nest space with a check of their next position vs the position of the boundaries of the space.

The length of the simulation space is measured in units (or AU). We approximated the sizes and distances of the ants as 5 units equal to 1 cm. We chose 5 units as the threshold for establishing contact to incorporate the interaction between the antennae of two ants. Contacts are also established in the areas surrounding the queen. The queen does not interact with the same worker twice in a row to avoid possible errors when determining contact between the queen and workers. If multiple workers are within range of the queen in a short time, this allows the queen to contact more of these workers.

Reviewer 3 suggests that:

“Figure S3 is used to demonstrate that the density of workers is kept almost constant independent of the size of the colony. Seeing this figure, I agree that the density in the regions where workers are staying seems to be kept almost constant. On the other hand, low-density regions of workers, also, are observed. Considering that the queen patrols over the whole area in the nest, I am afraid if it is appropriate, in defining the “density, to ignore the locally low density regions which decrease with the decrease of colony size. If we include these locally low-density regions in the definition, the spatial average of the worker-density increases linearly with the size, which does not fit the basic assumption of the present study.”

As stated in our results, the spatial distribution of workers around the queen remained constant. To emulate this, workers are evenly distributed in the simulation environment. Previous work by Franks et al[^5^](https://docs.google.com/document/d/1lQ9XFYXs0Uiyx-6kAr6Sie12st-BeXj1J_dVcBcoKgo/edit#bookmark=id.xv16x83t8ek6) also found that ants expanded their nest space proportional to the number of nestmates. Unlike the experimental set-up, the simulation nest size increased with colony size to maintain density and the spatial distribution of workers rather than altering the worker's movement in the colony to maintain the spatial distribution.

We understand that the reviewer found the section on the contact rates between the queen and workers hard to understand. For this, we apologise. We revised that section to make the definitions and explanations clear. Please find the revised section on page 16, Line 403:

“By logging the number of contacts that occurred during the simulation, various contact rates could be calculated. These are the queen contact rate, the per-worker contact rate and the contact rate between workers. The contact rate is defined as the number of contacts per unit time. Therefore, the queen contact rate is the rate at which the queen contacts workers per unit time. While the per-worker contact rate is the average contact rate of a worker in the colony. These values were calculated separately, with contacts being logged for the queen and individual workers. Theoretically, assuming an even distribution of contacts between workers, the per-worker contact rate is equivalent to the queen contact rate divided by the number of workers. However, the per-worker contact rate conveys the contact efficiency of the queen and therefore, the effectiveness of the queen's patrol behaviour.”

“Figure 7 shows various contact rates between the queen and workers as a function of the colony size. Figure 7A shows the overall contact rate for the queen increasing (black line) while the per-worker contact rate decreases (blue line). Despite the increased patrol effort by the queen (shown in Fig.4), the contact efficiency of the queen decreases with colony size. This is due to the insufficient increase in the queen contact rate. Distinctions were made for the contact rate during the rest cycles (Fig. 7B) and patrol cycles (Fig. 7C) of the queen. This was to demonstrate that the majority of the contacts by the queen were made when the queen was patrolling.”

Finally, reviewer 3 states:

“It is very interesting to see the authorsʼ scenario for the shift from the “ergonomic stage” to the “reproductive stage” according to the present results. However, I still wonder whether the realization of the shift from the “ergonomic stage” to the “reproductive stage” strongly depends on the parameter in the simulation or not. For example, if the values of delta, alpha and/or kappa, are set larger than the present ones, the negative feedback mechanism seems to work more remarkably, then, does the shift from the“ergonomic stage” to the“reproductive stage” still occur? Or, are all values set in the simulation supported by reality?”

The shift from the “ergonomic stage” to the “reproductive stage” is dependent on the parameters of the simulation insofar as they determine when the change occurs. The shift could primarily happen in 2 ways. Firstly, increasing beta and gamma would increase the rate at which workers develop their internal state. The increase would cause the change from the “ergonomic stage” to the “reproductive stage” to occur more quickly at smaller colony sizes when compared to the default values (see plot below). We increased beta and gamma to 10x larger, which caused a drastic decrease in the effectiveness of the queen’s patrol behaviour. It could be argued that the transition occurs at colony sizes larger than N=40 in this setup.

Secondly, changing epsilon and alpha (in eq. 2) would increase the sensitivity of the queen to increases in the worker's internal state. The increased sensitivity would likely cause the shift to occur more slowly at larger colony sizes. Kappa determines how much the internal state of the worker decreases during contact with the queen. A decrease in kappa would lead to less effective suppression during contact. We set the values to be in line with reality if appropriate. Explicitly, we set beta and gamma to increase the internal state of the workers at a pace which mirrored that reported by Kikuchi et al[^3^](https://docs.google.com/document/d/1lQ9XFYXs0Uiyx-6kAr6Sie12st-BeXj1J_dVcBcoKgo/edit#bookmark=id.tivf8jqsqaz3).
