# Supplementary material for: A real-time feedback system stabilises the regulation of worker reproduction under various colony sizes
Source: PLoS Comput Biol. 2023 Mar 24;19(3):e1010840. doi: 10.1371/journal.pcbi.1010840 (PMC10075462; doi:10.1371/journal.pcbi.1010840)
Supplement: Attachment — Submitted filename: Reviewer Response.docx [file pcbi.1010840.s014.docx]

# General Comments

We want to thank the reviewers for their comments on the manuscript and appreciate the feedback given. We are glad that we have been able to address the reviewers’ comments and improve the strength of our manuscript to be worthy of publication. We appreciate the comment from Reviewer 3 about one of our results and hope that we have addressed this appropriately.

We hope that you will find our response sufficient for the publication of our paper. We also hope this fortifies our manuscript and leads to the publication of our manuscript in this esteemed journal, PLOS Computational Biology.

# Response to Reviewer 3

We thank reviewer 3 for the feedback and would like to address their comments.

**Comment 1:**

I feel the mechanism for the result shown by the decreasing graph (blue line) in fig.8 is unclear. Why does the per-worker contact rate decrease with the colony size under the assumptions; i) the internal state change of workers does not affect the movement of workers, ii)the number density of workers is kept constant independent of the colony size? Because this result looks (at least for me) against intuition, the authors are desired to suggest some underlying mechanism.

**Comment 1 Response**

We have amended our manuscript to include our explanation of the decrease in the per-worker contact rate in Fig 8. Please find the revised part on Page 12, line 427.

‘The per-worker contact rate decreases for the same reason it decreases for the queen. The contact efficiency is lost at larger colonies because the agent's movement is insufficient to cover the space. However, for the queen, we can relate this to her behaviour because her internal state is related to her movement.

In comparison, the worker's movement stays the same at all times. Hence, the loss in per-worker contact rate between workers is likely more significant than the drop in per-worker contact rate with the queen. Although there are more workers than the queen and as the simulation ends once the queen has contacted every worker at least once, there is a more considerable drop in the per-worker contact rate with the queen in Fig 7A than in Fig 8. The workers do not have to have unique contact with other workers. In contrast, the queen does have unique contacts because she needs to address every worker individually.’

**Comment 2:**

This is a small but unignorable issue. In the explanation, in L269-270, of the last terms of eq. (2) and (3), delta(x_q-x_w), authors call this function a “delta function”. This is against the widely accepted definition of the delta function because zero point of the delta function is infinity.
The authors should call this term the “Kronecker’s delta” instead of a “delta function”
and should denote as delta_{x_q, x_w)}.

**Comment 2 Response**

We have amended our manuscript to change the term from a “delta function” to “Kronecker’s delta”. Please find the revised part on Page 7, line 267.

‘The term δ(r) denotes Kronecker's delta, i.e., its value is zero except when the distance between $x_{q}$ and $x_{w}$ is zero, then δ(r) = 1.’
